# Biomechanical investigation of the hybrid lumbar fixation technique with traditional and cortical bone trajectories in transforaminal lumbar interbody fusion: finite element analysis

**DOI:** 10.1186/s13018-023-04027-6

**Published:** 2023-07-31

**Authors:** Ying Huang, Abulikemu Maimaiti, Yiming Tian, Zhengrong Li, Alafate Kahaer, Paerhati Rexiti

**Affiliations:** 1 Xinjiang Medical University, Urumqi, China; 2 Department of Spine Surgery, The First Affiliated Hospital of Xinjiang Medical University, Urumqi, China; 3 Key Laboratory of High Incidence Disease Research in Xingjiang (Xinjiang Medical University), Ministry of Education, Urumqi, China; 4 Xinjiang Clinical Research Center for Orthopedics, Urumqi, China

**Keywords:** Cortical bone trajectory, Lumbar pedicle screws, Finite element analysis, Biomechanics

## Abstract

**Objective:**

To compare the biomechanical performance of the hybrid lumbar fixation technique with the traditional and cortical bone trajectory techniques using the finite element method.

**Methods:**

Four adult wet lumbar spine specimens were provided by the Department of Anatomy and Research of Xinjiang Medical University, and four L1–S1 lumbar spine with transforaminal lumbar interbody fusion (TLIF) models at L4–L5 segment and four different fixation techniques were established: bilateral traditional trajectory screw fixation (TT–TT), bilateral cortical bone trajectory screw fixation (CBT–CBT), hybrid CBT–TT (CBT screws at L4 and TT screws at L5) and TT–CBT (TT screws at L4 and CBT screws at L5). The range of motion (ROM) of the L4–L5 segment, von Mises stress of cage, internal fixation, and rod were compared in flexion, extension, left and right bending, and left and right rotation.

**Results:**

Compared with the TT–TT group, the TT–CBT group exhibited lower ROM of L4–L5 segment, especially in left-sided bending; the CBT–TT group had the lowest ROM of L4–L5 segment in flexion and extension among the four fixation methods. Compared with the CBT–CBT group, the peak cage stress in the TT–CBT group was reduced by 9.9%, 18.1%, 21.5%, 23.3%, and 26.1% in flexion, left bending, right bending, left rotation, and right rotation conditions, respectively, but not statistically significant (*P* > 0.05). The peak stress of the internal fixation system in the TT–CBT group was significantly lower than the other three fixation methods in all five conditions except for extension, with a statistically significant difference between the CBT–TT and TT–CBT groups in the left rotation condition (*P* = 0.017). In addition, compared with the CBT–CBT group, the peak stress of the rod in the CBT–TT group decreased by 34.8%, 32.1%, 28.2%, 29.3%, and 43.0% under the six working conditions of flexion, extension, left bending, left rotation, and right rotation, respectively, but not statistically significant (*P* > 0.05).

**Conclusions:**

Compared with the TT–TT and CBT–CBT fixation methods in TLIF, the hybrid lumbar fixation CBT–TT and TT–CBT techniques increase the biomechanical stability of the internal fixation structure of the lumbar fusion segment to a certain extent and provide a corresponding theoretical basis for further development in the clinic.

## Introduction

In contrast to cancellous bone, the cortical bone does not exhibit significant deformation and degeneration with increasing age [1]. The cortical bone trajectory (CBT) technique was first proposed by Santoni et al. in 2009 [2]. Compared with the conventional pedicle screw technique, it used the long axis of the pedicle as the screw path and designs the direction of the screw path to be forward and upward, slightly outwardly offsetting the screw to ensure that the screw was embedded with the lateral bone cortex of the pedicle [3, 4], increasing the axial tensile force by 30% and the screw torque by 1.7 times, with better mechanical properties [2, 5–9]. In addition, the CBT technique minimized the exposure area and reduced damage to the surrounding muscles and soft tissues of the spine, making it even more minimally invasive [10]. Meanwhile, if transforaminal lumbar interbody fusion (TLIF) was performed in patients with degenerative lumbar spine disease, compared with the traditional TT technique, patients with CBT technique had a shorter operative time and less intraoperative bleeding, while they were similar in terms of the accuracy of the postoperative screw position, the fusion rate, and the maintenance of the lordotic angles in the medium- and long-term follow-up observation of patients [11]. However, the CBT technique was also associated with a series of complications, such as intraoperative or postoperative fractures, dural tears, infections, and neurological injury [12–15]. In addition, the CBT technique had the following shortcomings: (1) the scope of nerve decompression was limited, especially in the presence of compression factors in the lateral recesses and intervertebral foramen; (2) the screw placement point of CBT split or fractured during decompression, making screw placement impossible; and (3) the CBT technique was contraindicated in patients with pars fracture [16–18].

Other studies have reported the use of a hybrid fixation technique in transforaminal lumbar interbody fusion.  This technique allowed for the shortening of the fixation level and achieved spinal stability without invading the adjacent segment [19]. However, there were still relatively few reports on the biomechanical properties of the hybrid fixation technique. In this study, the biomechanical properties of different lumbar internal fixation techniques have been evaluated by finite element analysis (FEA), especially the hybrid fixation technique in TLIF to provide a reliable and accurate mechanical theoretical basis for clinical application.

## Materials and methods

### Model development of the intact L1–S1 lumber spine

Construction and validation of the FE models were completed in the previous study [20]. The material properties were referred to the previous models [20]. The finite element model included five lumbar vertebrae, one sacrum, and five intervertebral disks, and each segment contained two endplates and seven ligaments. Cortical bone thickness was defined as 0.5–1 mm [21], and two cartilage endplates of 1 mm thickness were attached to the upper and lower surfaces of the vertebral body, with the nucleus pulposus simulating a fluid, incompressible substance that occupied 44% of the disk volume [22]. The diameter of the TT screw was 6.0 mm, with a length of 45 mm; while the CBT screw had a diameter of 5.0 mm and a length of 35 mm.

### Construction of surgical models

**Fig. 1:**
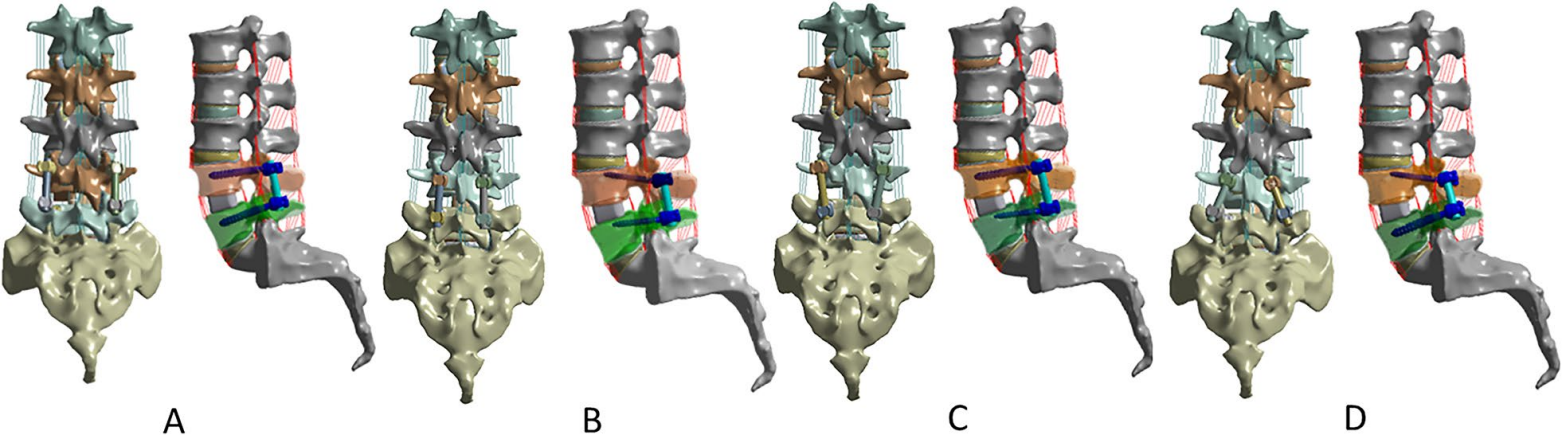
FE models of L1–S1 lumbar spine with TLIF at the L4–L5 segment with four different fixation techniques. **A** TT at L4 and L5 (TT–TT) [20]; **B** CBT at L4 and L5 (CBT–CBT) [20]; **C** TT screws at L4 and CBT at L5 (TT–CBT) [20]; **D** CBT at L4 and TT screws at the L5 (CBT–TT) [20]

The TLIF procedure was performed in Mimics 17.0 (Materialize, Leuven, Belgium) with decompression and fusion of the L4–5 vertebrae. The L4–L5 vertebral body was then internally fixed with the following combinations of internal screws: (1) TT–TT group, TT at both L4 and L5 segments (Fig. 1A) [20]; (2) CBT–CBT group, CBT at both L4 and L5 segments (Fig. 1B) [20]; (3) TT–CBT group, TT at L4 and CBT at L5 (Fig. 1C) [20]; (4) CBT -TT group, CBT at L4 and TT at L5 (Fig. 1D) [20]. The CBT screw entry point was located at the lateral point of the pedicle isthmus, and the screw was placed in the 5-point direction on the left side of the pedicle and in the 7-point direction on the right side, using bell-face positioning. The cortical bone screws were inserted 10° laterally in the transverse plane and 25° cranially in the sagittal plane [2].

### Boundary and loading conditions

Completely fixing and constraining the S1 lower endplate, a reference point was established in the upper surface center region of the L1 vertebra, which used a coupling to the upper surface region. A compressive load of 400 N and a torque of 7.5 Nm were applied to this reference point to simulate flexion, extension, left and right lateral bending, and left and right rotation, respectively. The ROM of L4–L5 segment, cage stress, fixation stress, and rod stress were analyzed using Ansys Workbench 19.1 (Ansys, USA).

### Statistical methods

Statistical methods SPSS 27.0 software was used to analyze and process the data. The means of quantitative data were expressed as mean ± standard deviation. Paired t-test was used for the analysis of variance. All results were considered statistically significant at *P* < 0.05.

## Results

### Range of motion of the L4–L5 Segment

**Fig. 2:**
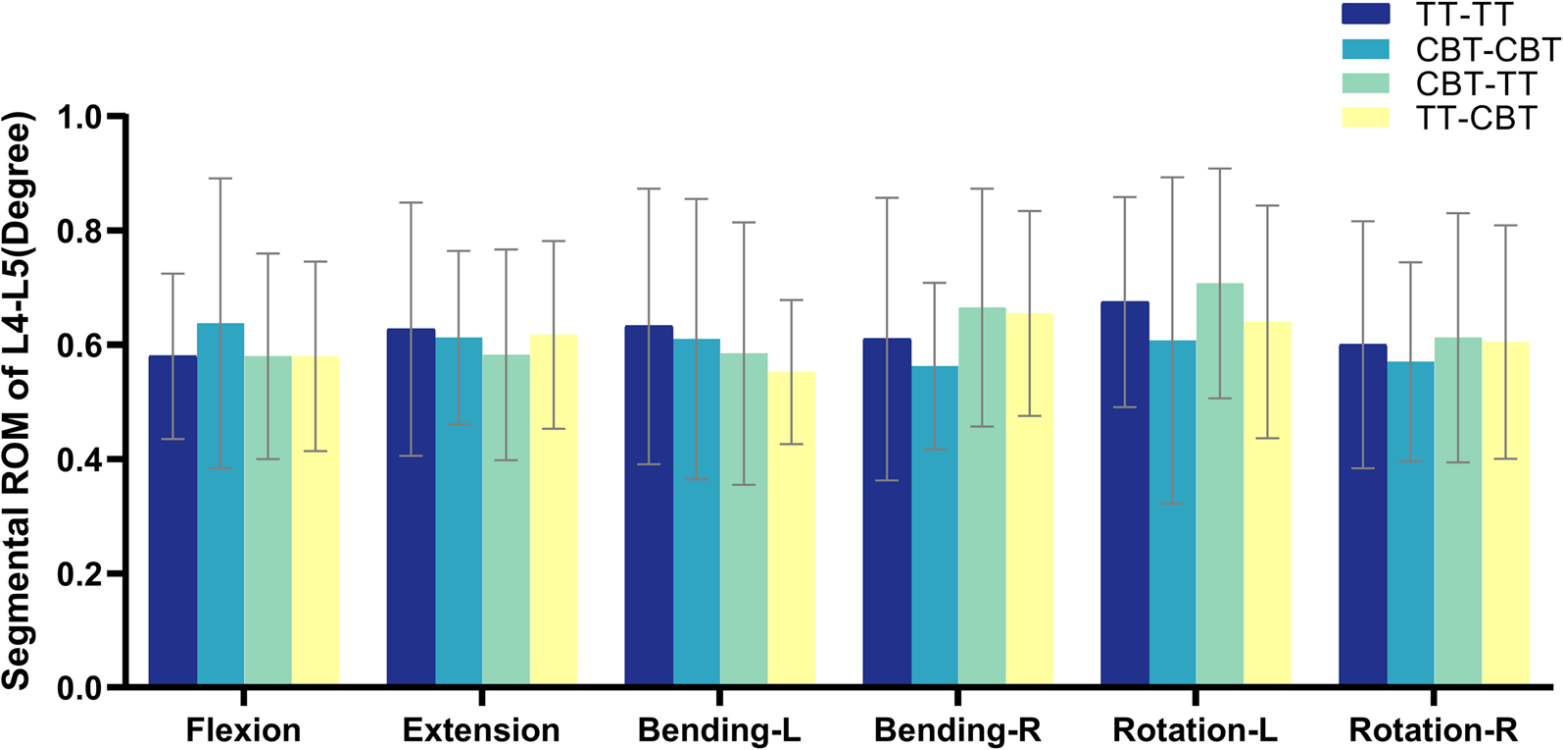
ROM of L4–L5 segment in four fixation techniques in six working conditions

The ROM of L4–L5 segment was shown in Fig. 2. Compared with the TT–TT group, the ROM of the CBT–CBT group increased by 9.9% in flexion and decreased by 2.4%, 3.6%, 7.8%, 10.0%, and 5.0% in extension, left bending, right bending, left rotation, and right rotation, respectively. ROM in the TT–CBT group increased by 7.4% and 0.8% under right bending and right rotation, as compared with the TT–TT group, and decreased by 1.6%, 12.6%, and 5.2% under extension, left bending, and left rotation, respectively; and increased by 0.8%, 16.4%, 5.3%, and 6.1% under extension, right bending, left rotation, and right rotation, as compared with the CBT–CBT group, respectively, and decreased by 9.0% and 9.4% under flexion and left bending decreased by 9.0% and 9.4%, respectively. The ROM in the CBT–TT group increased by 9.0%, 4.8%, and 2.1% under right bend, left rotation, and right rotation, respectively, compared to the TT–TT group, and decreased by 7.2% and 7.5% under extension and left bending, respectively, and was the same in both groups under flexion; increased by 18.2%, 16.5%, and 7.5% under right bending, left rotation, and right rotation, respectively, compared to the CBT–CBT group, and decreased by 9.0%, 4.9%, and 4.1% under flexion, extension, and left bending, respectively; in left bending, right bending, left rotation, and right rotation increased by 5.9%, 1.5%, 10.5%, and 1.2%, respectively, compared to the TT–CBT group, and decreased by 5.7% under extension, which was the same in both groups under flexion. There was a statistically significant difference between the CBT–CBT group and the TT–CBT group in the right bending condition (*P* = 0.031).

### Cage stress

**Fig. 3:**
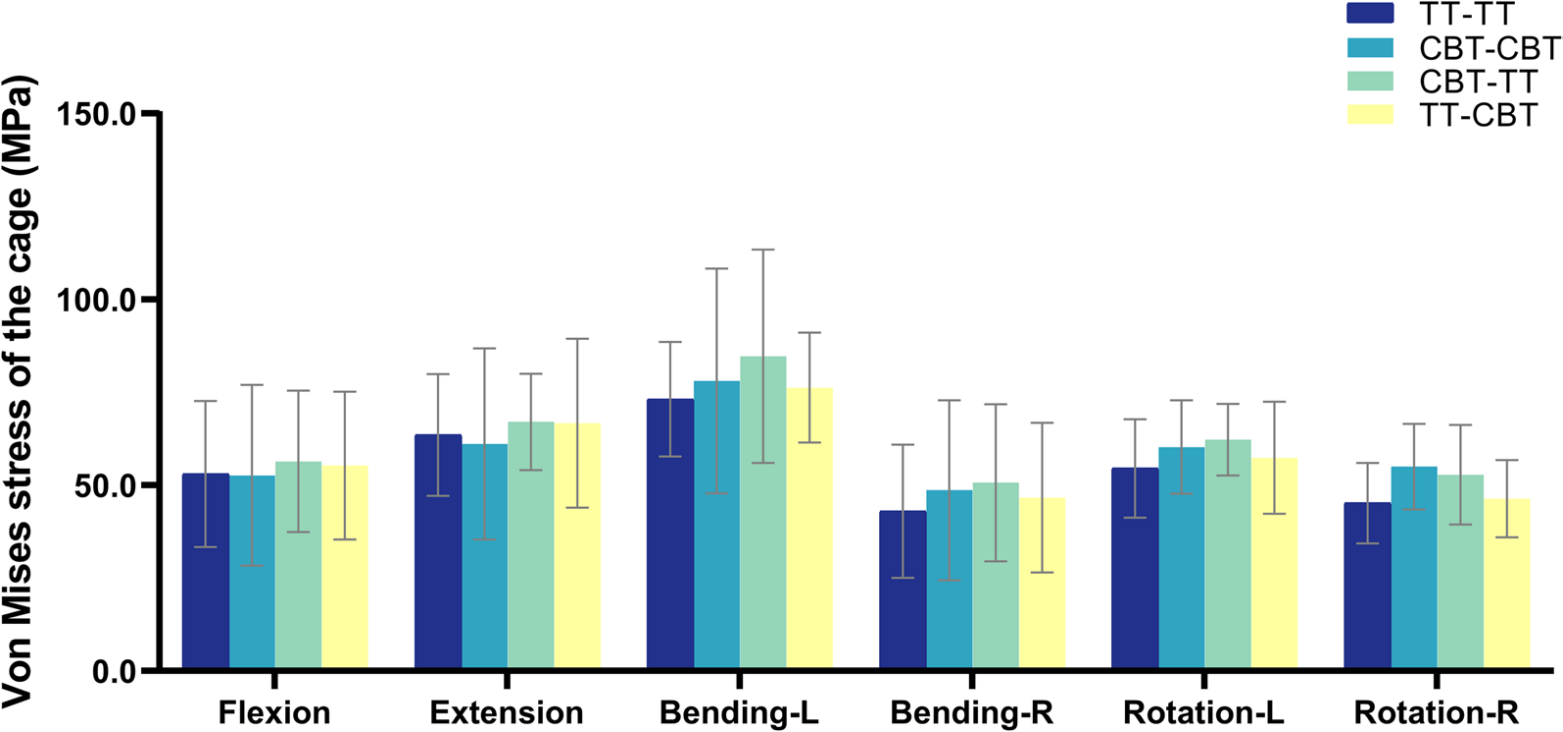
von Mises stress of the cage in four fixation techniques in six working conditions

The von Mises stress of the cage was displayed in Fig. 3. Compared with the TT–TT group, the mean value of peak cage stress in the CBT–CBT group increased by 6.8%, 13.1%, 10.5%, and 21.7% under left bending, right bending, left rotation, and right rotation, respectively, and decreased by 0.7% and 3.8% under flexion and extension, respectively. The mean value of peak cage stress in the TT–CBT group increased by 4.2%, 5.0%, 4.3%, 8.5%, 5.2%, and 2.5% compared with the TT–TT group under the six working conditions of flexion, extension, left and right bending, and left and right rotation, respectively; it increased by 4.9% and 9.1% compared with the CBT–CBT group under flexion and extension, respectively, and decreased by 2.3%, 4.0%, 4.8%, and 15.7% under left bending, right bending, left rotation, and right rotation, respectively. The mean value of peak cage stress in the CBT–TT group increased by 6.3%, 5.5%, 15.8%, 17.8%, 14.2%, and 16.8% in the six working conditions, respectively, compared with the TT–TT group, and increased by 2.1%, 0.5%, 11.1%, 8.5%, 8.5%, and 14.0% compared with the TT–CBT group; it increased by 7.1%, 9.7%, 8.5%, 4.1%, and 3.3% in flexion, extension, left bending, right bending, and left rotation increased by 7.1%, 9.7%, 8.5%, 4.1%, and 3.3%, respectively, and decreased by 4.0% under right rotation compared with the CBT–CBT group. Statistically significant differences existed between the TT–TT and CBT–TT groups in right bending conditions (*P = 0.029*), and between the TT–TT and CBT–CBT groups in left rotation (*P = 0.002*) and right rotation conditions (*P=0.046*).

### Stress of the internal fixation system

**Fig. 4:**
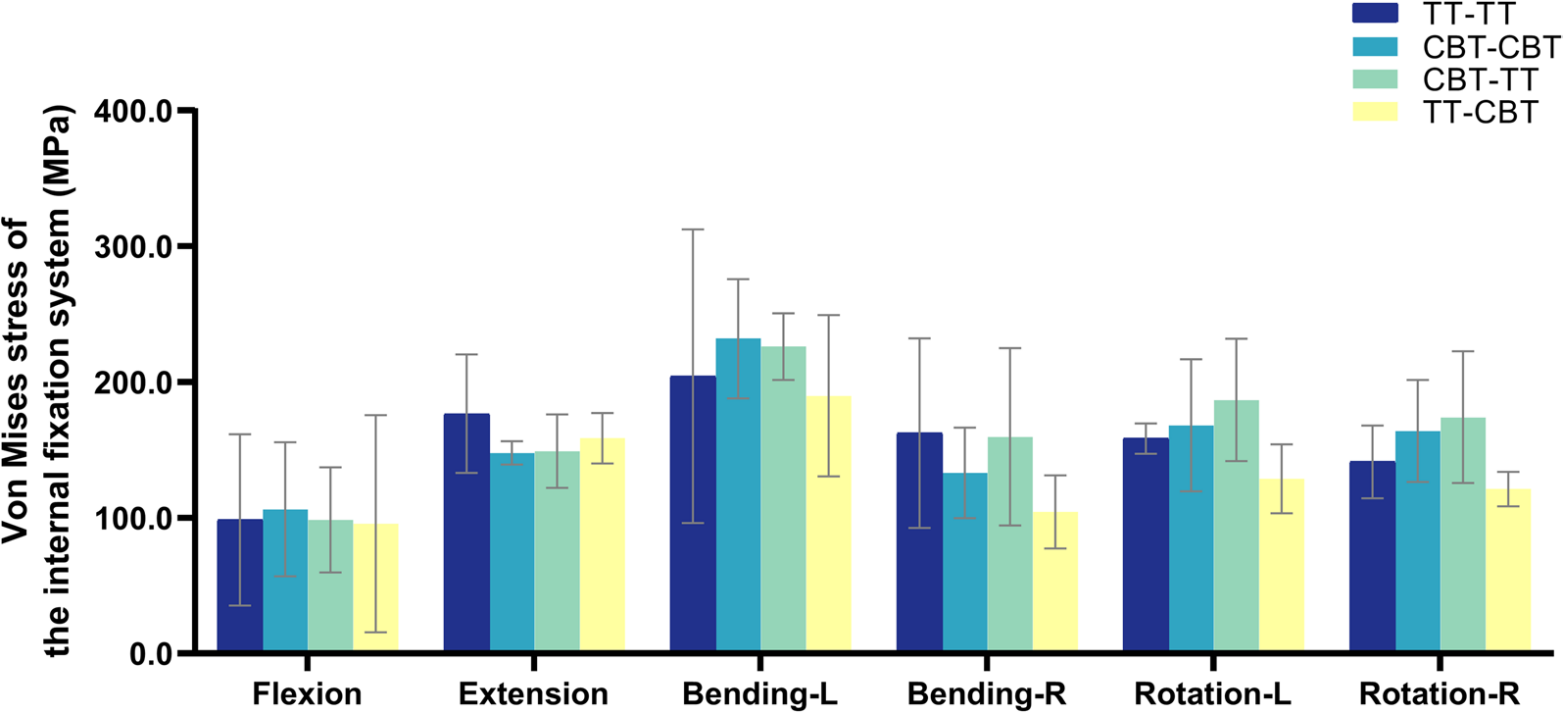
von Mises stress of the internal fixation system in four fixation techniques in six working conditions

The von Mises stress of the internal fixation system was displayed in Fig. 4. The smallest average value of peak stress in the internal fixation system under the six working conditions was the TT–CBT group. Compared with the TT–TT group, the mean value of peak internal fixation stress in the CBT–CBT group increased by 7.9%, 13.6%, 6.0%, and 16.2% under flexion, left bending, left rotation, and right rotation, respectively, and decreased by 16.3% and 18.0% under extension and right bending, respectively. The mean value of the peak stress of the internal fixation system in the TT–CBT group decreased by 2.8%, 10.2%, 7.0%, 35.7%, 18.7%, and 14.2% compared with the TT–TT group under the six working conditions of flexion, extension, left bending, right bending, left rotation, and right rotation, respectively; it increased by 7.3% compared with the CBT–CBT group under extension, and decreased by 9.9%, 18.1%, 21.5%, 23.3%, and 26.1% under flexion, left bending, right bending, left rotation, and right rotation, respectively. The mean value of peak stress of the internal fixation system in the CBT–TT group increased by 0.1%, 10.7%, 17.9%, and 23.2% under flexion, left bending, left rotation, and right rotation, respectively, and decreased by 15.6% and 1.7% under extension and right bending, respectively, compared with the TT–TT group; increased by 0.8%, 20.0%, 11.1%, and 6.1% under extension, right bending, left rotation, and right rotation, respectively, compared with the CBT–CBT group, and decreased by 7.2% and 2.5% under flexion and left bending, respectively; increased by 3.0%, 19.0%, 52.8%, 44.9%, and 43.6% under flexion, left bending, right bending, left rotation, and right rotation, respectively, and decreased by 6.0% under extension compared with the TT–CBT group. There was a statistically significant difference between the CBT–TT group and the TT–CBT group in the left rotation condition (*P* = 0.017).

### Stress of the rod

**Fig. 5:**
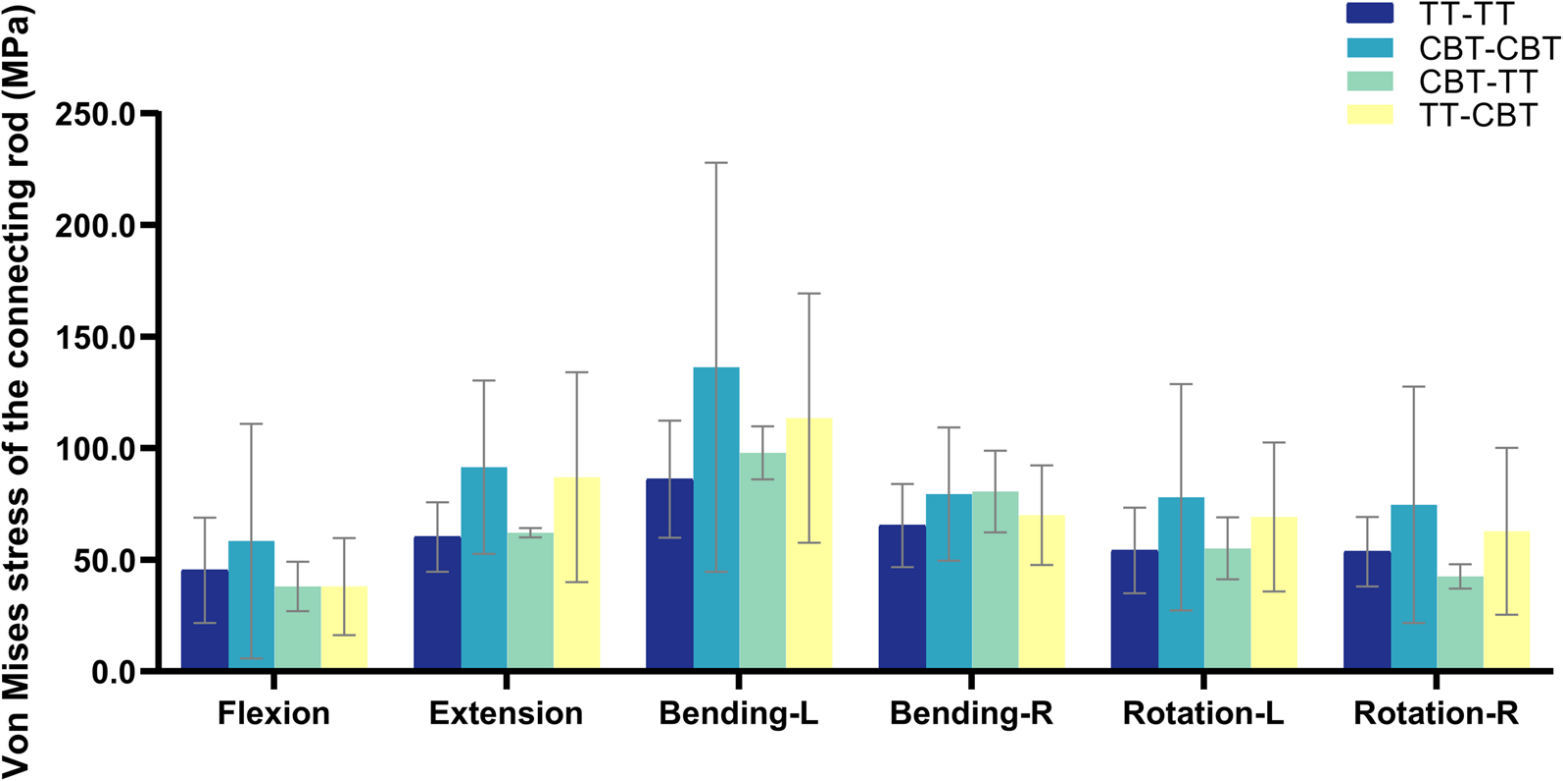
von Mises stress of the rod in four fixation techniques in six working conditions

The von Mises stress of the rod was displayed in Fig. 5. The largest average value of the peak stress of the rod under the six working conditions was in the CBT–CBT group. The mean value of peak stress of rod in the TT–CBT group increased by 44.3%, 31.9%, 7.2%, 27.6%, and 17.1% under extension, left bending, right bending, left rotation, and right rotation, respectively, and decreased by 16.1% under flexion compared with TT–TT group; in the six working conditions of flexion, extension, left bending, right bending, left rotation, and right rotation, it was reduced by 34.9%, 4.9%, 16.7%, 11.9%, 11.3%, and 15.9%, respectively. Compared with the TT–TT group, the average value of the peak stress of the rod in the CBT–CBT group increased by 28.9%, 51.8%, 58.3%, 21.7%, 43.9%, and 39.2% under the six working conditions of flexion, extension, left bending, right bending, left rotation, and right rotation, respectively. The mean value of the peak stress of the rod in the CBT–TT group increased by 3.1%, 13.7%, 23.4%, and 1.8% under extension, left bending, right bending, and left rotation, respectively, and decreased by 16.0% and 20.6% under flexion and right rotation, respectively, compared with the TT–TT group; increased by 1.4% under right bending compared with the CBT–CBT group, and decreased by 34.8%, 32.1%, 28.2%, 29.3%, and 43.0% under flexion, extension, left bending, left rotation, and right rotation, respectively; and increased by 0.1% and 15.1% under flexion and right bending, respectively, and decreased by 28.6%, 13.8%, 20.2%, and 32.2% under extension, left bending, left rotation, and right rotation, respectively, compared with the TT–CBT group.

## Discussion

The pedicle screw fixation technique, which improves fixation strength and fusion rates, has become the gold standard for spinal fusion [23, 24]. However, as the population ages, the number of patients with osteoporosis is constantly increasing, and various spinal disorders caused by osteoporosis are also growing [25]. In patients with osteoporosis, the decrease in bone density of the pedicle and vertebral body, as well as the enlargement of the trabecular meshwork, significantly reduces the holding power of pedicle screws during lumbar pedicle internal fixation. This results in decreased resistance to screw extraction and cyclic flexion, leading to loosening and failure of internal fixation [26]. Therefore, achieving strong internal fixation of the lumbar spine in patients with osteoporosis is a major challenge for spine surgery clinics [25].

### Overview of the hybrid lumbar TT and CBT fixation technique

The CBT technique is a superior alternative to traditional pedicle screws in clinical applications. Santoni et al. [2] performed experiments on the strength of CBT screw fixation using osteoporotic cadaver specimens and demonstrated that the axial pullout resistance of CBT was improved by more than 30% compared to TT screws. Calvert et al. [27] investigated the pullout strength of TT screws and CBT screws in comparison with 10 L3–L4 segmental lumbar spine samples and found that the axial pullout force of CBT screws could be increased by more than 60% compared to conventional TT screws. However, there are certain limitations in the clinical application of CBT technology. According to Alafate et al. [28], conventional transforaminal lumbar interbody fusion (TLIF) often required decompression of the lateral recess of the lower lumbar spine, which could affect the positioning of CBT screws. Paerhati et al. [29, 30] observed that the insertion point of cortical bone screws was closer to the spinal canal and nerves compared to conventional pedicle screws. They also noted that incorrect angle insertion of screws could cause damage to the adjacent nerve tissue and the nucleus pulposus tissue in the intervertebral space above the fused segment, which could lead to adjacent segment degeneration (ASD). Prior research [2, 3, 16, 28] has demonstrated that the hybrid lumbar fixation technique offered superior fixation strength and was less invasive compared to using either TT or CBT techniques alone. Takata et al. [31] applied a hybrid CBT–TT fixation technique in six patients with degenerative lumbar spondylolisthesis. This approach resulted in a significant reduction in the length of the surgical incision (5–6 cm), minimized intraoperative stripping of the paravertebral muscles, and decreased the risk of postoperative complications. Ueno et al. [32] presented a new surgical method for posterior corrective fusion using a hybrid fixation technique in patients with osteoporosis, which yielded favorable clinical outcomes. As clinical interest in minimally invasive techniques continues to rise, the hybrid lumbar fixation technique offers a more flexible and diverse option for lumbar fusion surgeries, and holds great potential for clinical application. However, there is a relatively limited number of research studies that have analyzed the mechanical properties of the hybrid lumbar fixation technique, and in articles that use finite element analysis, the modeling process is comparatively simple. Therefore, it is essential to develop a comprehensive finite element analysis model of the lumbar spine bones and ligamentous tissues, which encompasses various critical ligaments, small joints, intervertebral disks. This will enable a more objective analysis of the biomechanical performance data of the hybrid lumbar fixation technique in TLIF across multiple osteoporotic anatomical specimens.

### Finite element modeling characteristics

Finite element analysis, being a dependable and replicable simulation calculation method, has gradually played an indispensable role in the study of spinal diseases and implant biomechanics. The use of 3D finite element method to create a digital model can more accurately simulate various complex working conditions of the spine and calculate the stress distribution under different loads. This approach can aid in exploring the damage mechanism and reduce the time and effort required for repeated mechanical tests. Matsukawa et al. [25] conducted a biomechanical analysis using finite element analysis and confirmed that the hybrid fixation technique offered superior fixation strength in each plane of motion compared to the TT and CBT techniques. For patients with osteoporosis, the hybrid lumbar fixation technique may serve as an effective alternative to posterior fusion. However, since 3D finite element models are constructed based on scanned data, the accuracy of data identification and the authenticity of the structure and material are crucial factors that can impact the validity of the models. Thus, the ability to simulate the real characteristics of human tissue structure more precisely and comprehensively will significantly impact the credibility of the study results.

In comparison with previous studies, the finite element model developed in this study offered the following advantages: (1) the L4–L5 lumbar segments were commonly affected by lumbar degeneration and other disorders, whereas previous studies mostly focused on the L3–L4 lumbar segments. Therefore, the selection of L4–L5 lumbar segments in this study made the finite element model more clinically relevant to the common sites of lumbar degeneration and other disorders. (2) The finite element model developed in this study offered stronger model integrity, a higher level of detail, and more complex modeling compared to previous studies. It was a comprehensive lumbar spine model, which included all lumbar spine structures from L1 to S1. To enhance the accuracy and realism of the model, the mechanical properties of various ligaments, such as the anterior longitudinal ligament, posterior longitudinal ligament, ligamentum flavum, supraspinous ligament, interspinous ligament, intertransverse process ligament, joint capsule, and even articular cartilage, were defined individually. The complexity of modeling resulted in an exponential increase in computational workload. (Four FE models were established for each specimen. TT–TT group had 2,173,750 elements and 3,358,666 nodes; CBT–CBT group had 2,320,362 elements and 3,636,131 nodes; CBT–TT group had 2,157,039 elements and 3,354,904 nodes; and TT–CBT group had 2,173,750 elements and 3,358,666 nodes.) The majority of prior research only characterized the properties of the bone and disk structures in the L3–L4 lumbar segments, without fully accounting for the comprehensive force distribution throughout the lumbar spine and the intricate anatomical structures of the ligaments and synovial joints encompassing the lumbar spine. These factors had a significant influence on the modeling and analysis outcomes. (3) Selecting a larger number of experimental samples resulted in a relatively more representative sample. Specifically, this study used four osteoporotic lumbar spine anatomical specimens for scanning and modeling, whereas previous studies often only selected a single sample [33–35]. (4) The specimens chosen for this study were derived from osteoporotic anatomical specimens, which was in line with the potential clinical application of the hybrid lumbar fixation technique. Conversely, previous studies may use CT data from patients with normal bone density due to limitations [35–37], or the source of the data was not described in the study methodology [38]. (5) The modeling process fully considered all the details of the clinical TLIF procedure, including the extent of decompression, the insertion of the cage, and the placement of fixation screws and rod. Overall, the 3D finite element analysis model created in this study closely approximated the actual mechanical environment of the human lumbar spine, yielding more precise and dependable data to further guide the clinical practice.

### Clinical implications of this study

TLIF has been widely used for the treatment of degenerative lumbar spine diseases due to its advantages such as less invasive and good postoperative spinal stability. This procedure has shown good clinical application with complete decompression, short operative time, low bleeding, little damage to the spine, quick postoperative recovery, low complication rate, and preservation of most of the posterior vertebral column structures [39, 40]. However, compared to the TT–TT and CBT–CBT techniques, biomechanical analysis of the stress profile of fused segments in TLIF with hybrid CBT–TT and TT–CBT fixation techniques has been less reported in the literature, mostly as solid studies of human anatomical specimens, without the use of FEA [19, 31]. In this study, the biomechanical performance differences between traditional single or hybrid lumbar fixation techniques in TLIF using the FE model have been further investigated.

#### ROM of L4–L5 segment

The mobility of the fused segment often served as an indicator of the stability of the internal fixation system, and the finite element analysis conducted by Wu et al. [41] demonstrated that the stability of the fixed segment was greater in the CBT model than in the TT model, which was consistent with the results reported by Matsukawa et al. [3].

Previous biomechanical studies [16, 42, 43] shown that, in comparison with TT–TT, CBT–CBT exhibited adequate stiffness in flexion and extension conditions, but inferior mechanical performance in lateral bending and rotation conditions. The present study demonstrated that, in terms of vertebral stability, the CBT–CBT group outperformed the TT–TT group in extension (CBT–CBT: 0.613 ± 0.151°, TT–TT: 0.628 ± 0.222°), left bending (CBT–CBT: 0.610 ± 0.245°, TT–TT: 0.633 ± 0.241°), right bending (CBT–CBT: 0.563 ± 0.146°, TT–TT: 0.610 ± 0.247°), left rotation (CBT–CBT: 0.608 ± 0.285°, TT–TT: 0.675 ± 0.184°), and right rotation (CBT–CBT: 0.570 ± 0.174°, TT–TT: 0.600 ± 0.216°) conditions, with the exception of the flexion (CBT–CBT: 0.638 ± 0.254°, TT–TT: 0.580 ± 0.144°) condition. This may be attributed to various factors, such as the size of the screws used in the study, the trajectory of the screw placement, the type and completeness of the model constructed, and the different vertebrae subjected to compressive loads and torques. These factors required further analysis and investigation in subsequent mechanical tests conducted on human anatomical or animal solid specimens.

In the present study, the CBT–TT group exhibited the lowest range of motion (ROM) of the L4–L5 segment in flexion and extension compared to the other three groups. However, the ROM of L4–L5 segment was greater in both lateral bending and rotation, indicating that the ROM of L4–L5 segment in the CBT–TT group was superior in flexion and extension, but inferior in lateral bending and rotation. Compared to the TT–TT group, the TT–CBT group exhibited a reduced ROM of L4–L5 segment, particularly in left lateral bending (the TT–CBT group: 0.553 ± 0.126°, 12.6% lower than the TT–TT group: 0.633 ± 0.241°); however, there was no statistically significant difference when compared to the CBT–CBT group. These findings were consistent with previous studies on the biomechanical properties of the hybrid fixation technique using finite element analysis [28, 37], which suggested that the stability of the vertebral body formed by the TT–CBT group was enhanced, resulting in improved intersegmental stability.

#### Cage stress

The conventional TLIF procedure involves segmental decompression arthrodesis, interbody fusion implantation, and posterior pedicle screw fixation [44]. The intervertebral fusion cage is used in this procedure to restore intervertebral height, improve the lumbar spine alignment, and maintain local stability. Nonetheless, if the cage is excessively inserted into the intervertebral space, it may cause axial traction on the nerve roots, resulting in symptoms such as postoperative lower extremity numbness, pain, and foot drop [45]. Previous studies reported a high prevalence of cage subsidence following TLIF for lumbar degenerative conditions [46], and the sinking of the cage into the vertebral body was a complication that may occur after lumbar interbody fusion [47–49]. Numerous authors suggested that various types of internal fixation were crucial for preserving the stability of the surgical segment and minimizing the occurrence of cage subsidence [50]. Furthermore, osteoporosis was a risk factor for cage subsidence, and patients with this condition were more susceptible to postoperative cage subsidence due to bone destruction, significantly diminished strength of the endplate and vertebral bone, and the inability of the endplate to withstand greater stress [51, 52]. According to Tempel et al. [53], decreased bone mineral density was a highly sensitive (78.3%) and specific (63.2%) independent risk factor for cage subsidence. Finite element and autopsy studies demonstrated that individuals with osteoporosis exhibited reduced lumbar endplate disruption loads [54, 55] and were more vulnerable to cage settling [56]. Cage stress has not been extensively discussed in previous studies [28, 31, 33]. Related studies further confirmed that the greater the cage stress, the higher the subsidence rate [57]. Among the four screw placement methods evaluated in this study, the mean value of the peak cage stress in the CBT–TT group was the largest in all five working conditions except for the right bending, suggesting that the CBT–TT group may have a larger cage subsidence rate. However, since the difference in data between this group was not statistically significant, further confirmation through subsequent experiments was necessary. Furthermore, the CBT–CBT group exhibited the lowest average peak cage stress under flexion and extension conditions, while the TT–TT group demonstrated the lowest average peak cage stress under bending and rotation conditions. The decrease in cage stress implied that fusion stress was distributed by surrounding tissues, such as the vertebral body, rather than being concentrated in one area. The study revealed that the hybrid fixation TT–CBT group exhibited a slightly lower mean value of peak cage stress compared to the CBT–CBT group. This was particularly evident in the left bending (TT–CBT: 76.211 ± 14.775 MPa, CBT–CBT: 78.040 ± 30.268 MPa), right bending (TT–CBT: 46.671 ± 20.133 MPa, CBT–CBT: 48.638 ± 24.183 MPa), left rotation (TT–CBT: 57.351 ± 15.062 MPa, CBT–CBT: 60.251 ± 12.559 MPa), and right rotation (TT–CBT: 46.332 ± 10.385 MPa, CBT–CBT: 54.986 ± 11.491 MPa) conditions, with reductions of 2.3%, 4.0%, 4.8%, and 15.7%, respectively. Additionally, the reductions were slightly increased in the flexion and extension conditions compared to the CBT–CBT group. This suggested that the TT–CBT group had better resistance in both lateral bending and rotational movements and was less likely to cause cage settling. It could be inferred from the statement that the group undergoing TT–CBT exhibits superior resilience to both lateral flexion and rotational motion, thereby reducing the likelihood of cage settling [58].

#### Internal fixation system stress

Wu et al. [41] used finite element analysis to investigate the distribution of stress within the vertebral kinematic unit when using CBT screw fixation. Their findings revealed that the region of maximum stress distribution within the kinematic unit varied under different working conditions, but was primarily located in the screw rod. Conversely, the maximum stress experienced by the vertebral body was relatively minor. The distribution of stress depicted herein was indicative of the substantial restraining impact of the screw and internal fixation mechanism on the inclination of the vertebral unit to shift, thereby leading to the concentration of stress within the rod. The results showed that the maximum stress of the CBT screw was less than that of the TT screw when the cage was placed, but the difference between the two was not significant. However, the findings of this study revealed that the average peak stress in the internal fixation system was marginally higher in the CBT–CBT group compared to the TT–TT group, particularly in instances of flexion, leftward bending, leftward rotation, and rightward rotation. The current investigation may be associated with the fact that it presented a more comprehensive and authentic simulation of the actual TLIF procedure. Four finite element models were constructed to eliminate the zygapophyseal joints on the one side of the decompressed segment in a random manner, while the other side was kept intact. Interestingly, in comparison with the TT–TT group, the mean value of the peak stress of the internal fixation system in the CBT–CBT group increased by 13.6% in the left bending and decreased by 18.0% in the right bending. The marked dissimilarity between the left and right bending may imply that the mechanical characteristics of the decompressed side were relatively inferior to those of the non-decompressed side under specific operative conditions. This discovery further implied that during TLIF surgery, the spinal anatomy should be safeguarded to the greatest extent possible in order to enhance stability while alleviating the lesion.

McLachlin et al. [59] found the “teeter-totter” phenomenon in their study of an early loosening screw model, in which the posterior end of the screw is secured in the bone cortex while the anterior end oscillates in the trabeculae. According to Alafate et al. [28], the von Mises stress distribution within the cancellous bone region of the TT–TT group was consistently greater than that of other fixation techniques under identical conditions. Therefore, they proposed that screw–bone interface failure was more prone to occur when TT or CBT screws were used alone. The data obtained from this study indicated that the hybrid fixation technique may decrease the von Mises stress in this area, particularly during flexion and extension. This finding further corroborated the conclusion drawn by Matsukawa et al. [25] through finite element analysis of the hybrid fixation technique.

**Fig. 6:**
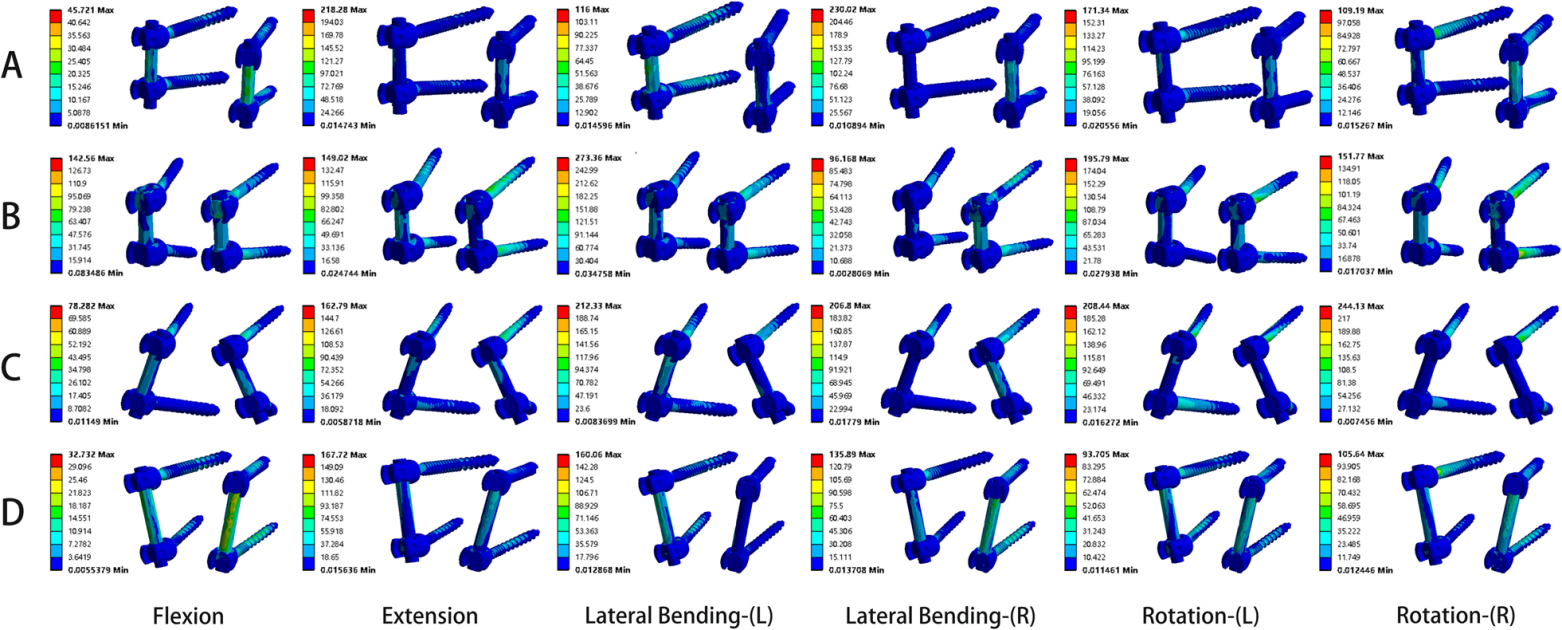
Stress nephograms of the internal fixation system of the four different implanted models in flexion, extension, left lateral bending, right lateral bending, left rotation, and right rotation, respectively. **A** Stress nephograms of TT–TT. **B** Stress nephograms of CBT–CBT. **C** Stress nephograms of CBT–TT. **D** Stress nephograms of TT–CBT

**Fig. 7:**
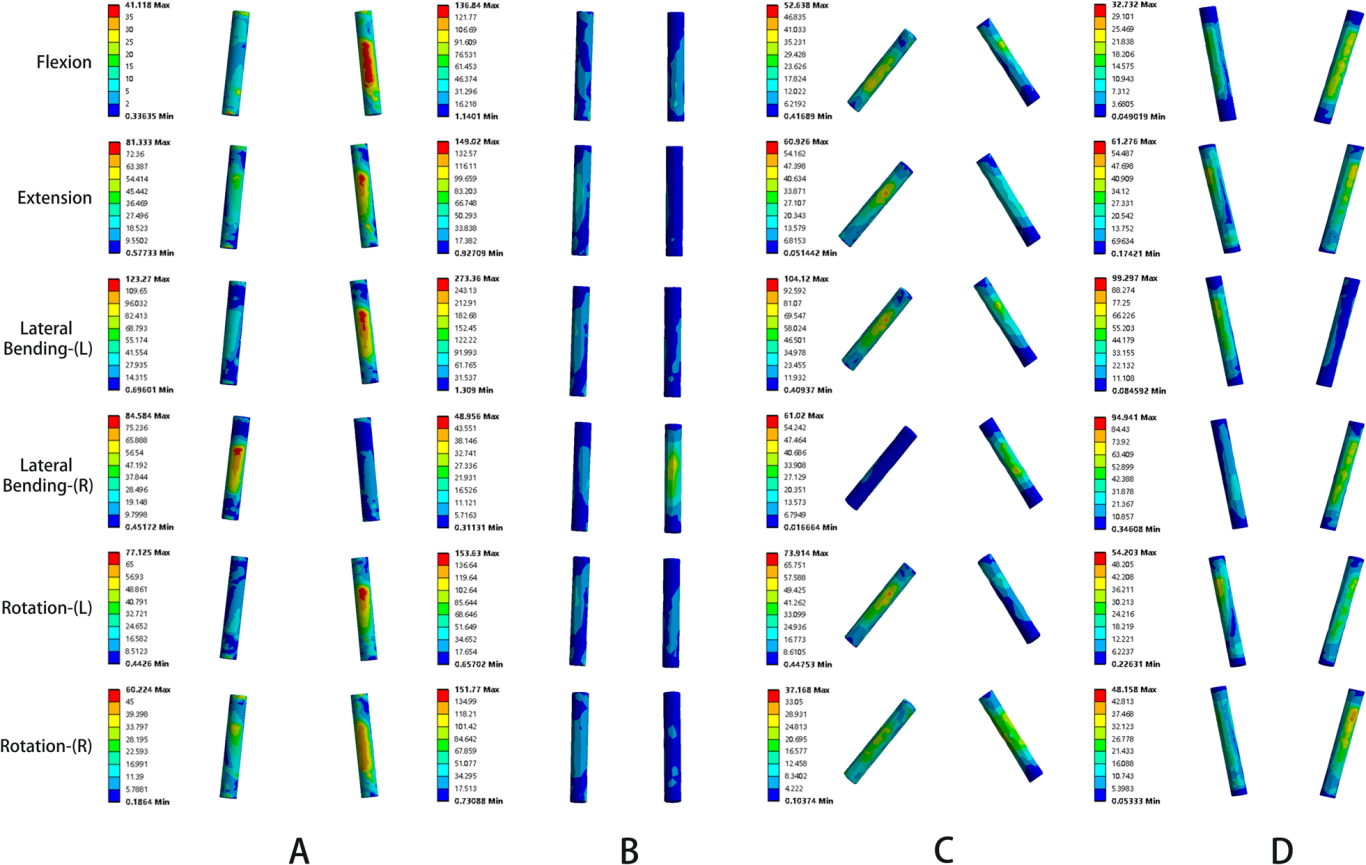
Stress nephograms of the rod of the four different implanted models in flexion, extension, left lateral bending, right lateral bending, left rotation, and right rotation, respectively. **A** Stress nephograms of TT–TT. **B** Stress nephograms of CBT–CBT. **C** Stress nephograms of CBT–TT. **D** Stress nephograms of TT–CBT

Newcomb et al. [60] suggested that high stress concentrations could lead to the failure of the fixation system. In this study, the mean value of the peak stress of the internal fixation system in the CBT–TT group was greater in both lateral bending and rotational conditions, which may suggest a greater risk of screw breakage. However, among the four fixation techniques, the CBT–TT group had a smaller mean value of peak stresses on the rod in lateral bending and rotational conditions, indicating that the stresses in the internal fixation system of the CBT–TT group were mainly concentrated on the screws rather than on the rod. Meanwhile, the mean values of peak stresses in the internal fixation system and the mean values of peak stresses in the rod were smaller in the CBT–TT group in both flexion and extension conditions, suggesting that the stress dispersion ability of the CBT–TT internal fixation method, compared with the flexion and extension conditions, was poorer in the lateral bending and rotation conditions, resulting in stress concentration (Figs. 6 and 7). Compared with the conventional fixation TT–TT and CBT–CBT groups, the mean value of peak stress in the internal fixation system of the hybrid fixation TT–CBT group was smaller than that of the TT–TT group in all six conditions and smaller than that of the CBT–CBT group in five conditions: flexion, left bending, right bending, left rotation, and right rotation, and only slightly larger in the extension condition. This was consistent with the findings of Alafate et al. [28]. This showed that the hybrid TT–CBT fixation technique could significantly reduce the peak stress of the internal fixation system in all directions of motion, resulting in a more uniform stress distribution and improving the strength and stability of the internal fixation system.

#### Rod stress

As research on the pedicle screw–rod fixation method continues to expand, fragmentation of the von Mises stresses on the screw–rod contact surface has been documented in the literature. Song et al. [61] illustrated that the screw fixation system, resembling a “crane” structure, revealed stress concentrations at the center of rotation where the screw came into contact with the rod. Meanwhile, Huang et al. [62] discovered that the von Mises stresses in the short rod model were concentrated at the tail end of the screw, posing a potential risk for structural damage in this particular region. These investigations demonstrated that the stresses within the internal fixation system were concentrated at the junction of the rod and the terminus of the screw, with the screw experiencing notable posterior concentration of stresses as well. This phenomenon potentially increased the susceptibility to rod and screw fracture. In this study, compared with the CBT–CBT group, the average value of the peak stress of the rod in the CBT–TT group decreased in all six working conditions, while the average value of the peak stress of the rod in the TT–CBT group was lower than that in the CBT–CBT group in the five working conditions of flexion, extension, left bending, left rotation, and right rotation, and only slightly increased in the right bending. The reduction in the mean value of the peak stress of the rod was particularly significant in the left bending (CBT–CBT: 136.359 ± 91.592 MPa; CBT–TT: 97.957 ± 11.826 MPa, reduced by 28.2%; TT–CBT: 113.599 ± 55.837 MPa, reduced by 16.7%) and right rotation(CBT–CBT: 74.688 ± 52.917 MPa; CBT–TT: 42.608 ± 5.484 MPa, reduced by 15.9%; TT–CBT: 62.829 ± 37.436 MPa, reduced by 32.2%) conditions (Fig. 7). This followed the results of a previous study [37]. In summary, the hybrid fixation technique showed a lower von Mises stress distribution in lateral bending and rotation of the rod compared to the single screw technique, which further suggested that the combined fixation technique had a lower risk of rod and screw breakage, which would further reduce the occurrence of postoperative complications due to excessive fatigue stress loading of the metal of the internal fixation system itself.

### Limitations of the this study

This study, however, still had certain limitations that were inherent to finite element analysis. First, the model established in this study was based on the scan data of human specimens. Compared with normal human bones, the scan data of cadaveric specimens were affected by the preservation conditions of specimens and sampling methods, which could potentially affect the analysis results. Secondly, despite the fact that this model had been modeled and processed as meticulously as possible, the impact of soft tissues such as muscles had been disregarded. Thirdly, this study simulated six kinds of motion states under different working conditions, while there were more complex compound motions and corresponding compound force states that had not been taken into account. Fourthly, the sample numbers were still relatively small, with variations in age, bone condition, and other factors among the specimens.

## Conclusions

In this study, the biomechanical characteristics of four fixation techniques, TT–TT, CBT–CBT, CBT–TT, and TT–CBT, were compared in transforaminal lumbar interbody fusion by using a three-dimensional finite element analysis modeling method. The aim of this research was to simulate the complete lumbar vertebrae and accessory structures as accurately as possible, in order to produce more realistic and credible data. Compared to TT–TT and CBT–CBT fixation methods, the hybrid CBT–TT and TT–CBT lumbar fixation techniques increased the biomechanical stability of the fixed structures within the lumbar fusion segment. This study took into consideration the pivotal factors pertaining to the biomechanical performance. However, further research data was required to provide additional support for the clinical application of the hybrid lumbar fixation technique.

## Data Availability

The data used to support the findings of this study are included within the article.
